# Laterality and region-specific tau phosphorylation correlate with PTSD-related behavioral traits in rats exposed to repetitive low-level blast

**DOI:** 10.1186/s40478-021-01128-3

**Published:** 2021-03-01

**Authors:** Georgina Perez Garcia, Rita De Gasperi, Miguel A. Gama Sosa, Gissel M. Perez, Alena Otero-Pagan, Dylan Pryor, Rania Abutarboush, Usmah Kawoos, Patrick R. Hof, Dara L. Dickstein, David G. Cook, Sam Gandy, Stephen T. Ahlers, Gregory A. Elder

**Affiliations:** 1 Research and Development Service, James J. Peters Department of Veterans Affairs Medical Center, 130 West Kingsbridge Road, Bronx, NY 10468 USA; 2 Department of Neurology, Icahn School of Medicine At Mount Sinai, One Gustave Levy Place, New York, NY 10029 USA; 3 Department of Psychiatry, Icahn School of Medicine At Mount Sinai, One Gustave Levy Place, New York, NY 10029 USA; 4 General Medical Research Service, James J. Peters Department of Veterans Affairs Medical Center, 130 West Kingsbridge Road, Bronx, NY 10468 USA; 5 Department of Neurotrauma, Naval Medical Research Center, 503 Robert Grant Avenue, Silver Spring, MD 20910 USA; 6 The Henry M. Jackson Foundation for the Advancement of Military Medicine, Inc., Bethesda, MD USA; 7 Nash Family Department of Neuroscience and Friedman Brain Institute, Icahn School of Medicine At Mount Sinai, One Gustave Levy Place, New York, NY 10029 USA; 8 Department of Geriatrics and Palliative Care, Icahn School of Medicine At Mount Sinai, One Gustave Levy, New York, NY 10029 USA; 9 Mount Sinai Alzheimer’s Disease Research Center and Ronald M. Loeb Center for Alzheimer’s Disease, Icahn School of Medicine At Mount Sinai, One Gustave Levy Place, New York, NY 10029 USA; 10 Department of Pathology, Uniformed Services University of Health Sciences, 4301 Jones Bridge Rd., Bethesda, MD 20814 USA; 11 Center for Neuroscience and Regenerative Medicine, Uniformed Services University of Health Sciences, 4301 Jones Bridge Rd., Bethesda, MD 20814 USA; 12 Geriatric Research Education and Clinical Center, VA Puget Sound Health Care System, 1660 S Columbian Way, Seattle, WA 98108 USA; 13 Department of Medicine, University of Washington, 1959 NE Pacific St, Seattle, WA 98195 USA; 14 Barbara and Maurice A. Deane Center for Wellness and Cognitive Health, and Mount Sinai NFL Neurological Care Center, Icahn School of Medicine At Mount Sinai, 5 East 98th Street, New York, NY 10029 USA; 15 Neurology Service, James J. Peters Department of Veterans Affairs Medical Center, Neurology Service (3E16), 130 West Kingsbridge Road, Bronx, NY 10468 USA

**Keywords:** Animal model, Behavior, Blast, Laterality, Rat, Tau, Traumatic brain injury

## Abstract

Military veterans who experience blast-related traumatic brain injuries often suffer from chronic cognitive and neurobehavioral syndromes. Reports of abnormal tau processing following blast injury have raised concerns that some cases may have a neurodegenerative basis. Rats exposed to repetitive low-level blast exhibit chronic neurobehavioral traits and accumulate tau phosphorylated at threonine 181 (Thr181). Using data previously reported in separate studies we tested the hypothesis that region-specific patterns of Thr181 phosphorylation correlate with behavioral measures also previously determined and reported in the same animals. Elevated p-tau Thr181 in anterior neocortical regions and right hippocampus correlated with anxiety as well as fear learning and novel object localization. There were no correlations with levels in amygdala or posterior neocortical regions. Particularly striking were asymmetrical effects on the right and left hippocampus. No systematic variation in head orientation toward the blast wave seems to explain the laterality. Levels did not correlate with behavioral measures of hyperarousal. Results were specific to Thr181 in that no correlations were observed for three other phospho-acceptor sites (threonine 231, serine 396, and serine 404). No consistent correlations were linked with total tau. These correlations are significant in suggesting that p-tau accumulation in anterior neocortical regions and the hippocampus may lead to disinhibited amygdala function without p-tau elevation in the amygdala itself. They also suggest an association linking blast injury with tauopathy, which has implications for understanding the relationship of chronic blast-related neurobehavioral syndromes in humans to neurodegenerative diseases.

## Introduction

Accumulation of abnormally phosphorylated tau is a feature of many human neurodegenerative diseases, collectively referred to as tauopathies [51]. In diseases such as Alzheimer's disease, the frontotemporal dementias and primary age-related tauopathy, these accumulations form a range of aggregated and oligomeric structures, with the best-known example being the neurofibrillary tangle (NFT) [51]. Tauopathy is a component of the recently formulated multiplex of genetic and molecular mechanisms that contribute to brain degeneration [91]. The mechanism(s) of abnormal tau hyperphosphorylation in these disorders remains incompletely understood, as does the role of various protein kinases and protein phosphatases that control tau phosphorylation.

Traumatic brain injury (TBI) is a risk factor for later development of neurodegenerative diseases that may have various underlying pathologies. Chronic traumatic encephalopathy (CTE) is one form of TBI-related dementia [18, 19, 34]. CTE is most often associated with repetitive mild TBI (mTBI) and is characterized pathologically by aggregation of hyperphosphorylated tau into NFTs, which occur in a characteristic pattern [68]. CTE can only be diagnosed definitively at autopsy but may be suspected clinically when a progressive cognitive/behavioral syndrome develops following repetitive mTBIs [34]. Recent ultrastructural evidence indicates that CTE tauopathy is distinct from other tauopathies, perhaps because in CTE novel moieties of either lipid or carbohydrate are incorporated into tau [30]. Computational modeling has further suggested that mechanical strain factors may explain why the pathology is most prominent in the depths of cortical sulci [37].

TBI is a long-standing concern in military life and has recently received increased attention because of the conflicts in Iraq and Afghanistan where 10–20% of deployed troops suffered a TBI [27]. While these TBIs occurred through various mechanisms, blast-related injuries resulting from exposures to mortars, artillery shells and improvised explosive devices were the major causes with repetitive mild injuries common [27]. Concerns also exist over potential adverse effects of subclinical blast exposures, which are common for many service members in combat as well as non-combat settings [29].

A number of reports have appeared of pathologically confirmed CTE in military veterans following blast injury [39, 59, 69, 76]. Tau pathology has also been prominent in veterans with chronic cognitive and behavioral syndromes following blast exposure even when classic CTE pathology was not present [46]. Positron emission tomography (PET) scanning using the tau ligand [^18^F]AV1451 has shown excessive ligand retention in veterans with a history of blast exposure who suffer from chronic cognitive and behavioral syndromes [21, 71, 84]. Others have noted increased plasma tau concentrations in recently deployed veterans with a history of TBI, that correlated with symptom severity [75]. While the relative risk of CTE following blast exposure is unknown, concern has been raised in the most recent veterans due to the frequency of repetitive mTBI [27].

Rats exposed to a repetitive low-level blast exposure protocol designed to mimic an mTBI or subclinical blast exposure develop a variety of cognitive and post-traumatic stress disorder (PTSD)-related behavioral traits [78, 81]. These traits develop in a delayed manner and become chronic and persistent being present for at least 1 year after blast exposure [78, 81].

Although not as well appreciated as in humans, rats and mice have been known to exhibit paw preference and hemispheric laterality for complex behavioral functions since the 1970s [20]. Hemispheric dominance in particular affects spatial memory in rodents [50, 89]. The brain dopaminergic system, which is disrupted by blast exposure [87] also shows well-established lateralization patterns [9, 14, 96]. In a prior study we reported that rats exposed to repetitive low-level blast exposure accumulate abnormal levels of phosphorylated tau (p-tau) with a laterality and regionally specific pattern [21]. Here we correlated levels of p-tau measured in this prior study [21] with behavioral parameters previously assessed in the same animals [81]. We report that p-tau levels correlate with blast-induced behavioral traits.

## Materials and methods

### Experimental animals

Adult male Long Evans hooded rats (250–350 g; 10 weeks of age; Charles River Laboratories International, Wilmington, MA, USA) were used. All studies involving animals were reviewed and approved by the Institutional Animal Care and Use Committees of the Walter Reed Army Institute of Research (WRAIR)/Naval Medical Research Center and the James J. Peters VA Medical Center. Studies were conducted in compliance with the Public Health Service policy on the humane care and use of laboratory animals, the NIH Guide for the Care and Use of Laboratory Animals, and all applicable Federal regulations governing the protection of animals in research.

### Blast overpressure exposure

Rats were exposed to overpressure injury using the WRAIR shock tube, which simulates the effects of air-blast exposure under experimental conditions [1]. The shock tube has a 30.48 cm circular diameter and is a 5.94 m-long steel tube divided into a 77.4-cm compression chamber that is separated from a 5.18 m expansion chamber. The compression and expansion chambers are separated by polyethylene Mylar™ sheets (Du Pont, Wilmington, DE, USA) that control the peak pressure generated. The peak pressure at the end of the expansion chamber was determined by piezoresistive gauges specifically designed for pressure–time (impulse) measurements (Model 102M152, PCB, Piezotronics, Depew, NY, USA).

Individual rats were anesthetized using an isoflurane gas anesthesia system consisting of a vaporizer, gas lines and valves and an activated charcoal scavenging system adapted for use with rodents. Rats were placed into a polycarbonate induction chamber, which was closed and immediately flushed with 5% isoflurane in air mixture for two minutes. Rats were placed into a cone shaped plastic restraint device and then placed in the shock tube. Movement was further restricted during the blast exposure using 1.5 cm diameter flattened rubber tourniquet tubing. Three tourniquets were spaced evenly to secure the head region, the upper torso and lower torso while the animal was in the plastic restraint cone. The end of each rubber tourniquet was threaded through a toggle and run outside of the exposure cage where it was tied to affix the animal firmly and prevent movement during the blast overpressure exposure without restricting breathing. Rats were randomly assigned to sham or blast conditions and were placed in the shock tube lying prone with the plane representing a line from the tail to the nose of the body in line with the longitudinal axis of the shock tube with the head placed more upstream. Further details of the physical characteristics of the blast wave were described previously [1]. Blast exposed animals received 74.5 kPa (equivalent to 10.8 psi, duration 4.8 ms, impulse 175.8 kPa*ms) exposures administered one exposure per day for three consecutive days. Control (sham-exposed) animals were treated identically including receiving anesthesia and being placed in the blast tube but did not receive a blast exposure. Within 10 days after the last blast or sham exposure animals were transported in a climate-controlled van from the WRAIR to the James J. Peters VA Medical Center (Bronx, NY, USA). Animals left in the morning from the WRAIR and arrived in the afternoon of the same day at the James J. Peters VA Medical Center, where all other procedures were performed.

### Animal housing

At the James J. Peters VA Medical Center, animals were housed at a constant 70–72° F temperature with rooms on a 12:12 h light cycle with lights on at 7 AM. All subjects were individually housed in standard clear plastic cages equipped with Bed-O'Cobs laboratory animal bedding (The Andersons, Maumee, Ohio, USA) and EnviroDri nesting paper (Sheppard Specialty Papers, Milford, NJ, USA). Access to food and water was ad libitum. Subjects were housed on racks in random order to prevent rack position effects. Cages were coded to allow maintenance of blinding to groups during behavioral testing.

### Behavioral testing

#### Elevated zero maze

The apparatus consisted of a circular black Plexiglas runway 121.92 cm in diameter and raised 76 cm off the floor (San Diego Instruments, San Diego, CA, USA). The textured runway itself was 5.08 cm across and divided equally into alternating quadrants of open runway enclosed only by a 1.27-cm lip and closed runway with smooth 15.24-cm walls. All subjects received a 5-min trial beginning in a closed arc of the runway. During each trial, subjects were allowed to move freely around the runway, with all movement tracked automatically by a video camera placed on the ceiling directly above the maze. Data were analyzed by ANYMAZE (San Diego Instruments, San Diego CA, USA) yielding measures of total movement time and distance for the entire maze, as well as time spent and distance traveled in each of the individual quadrants. From the quadrant data, measures of total open and closed arc times, latency to enter an open arc, total open arm entries and latency to completely cross an open arc between two closed arcs were calculated. Subject position was determined by centroid location.

#### Light/dark escape

A light/dark escape task was run in Versamax activity cages with opaque black Plexiglas boxes enclosing the left half of the interiors so that only the right sides were illuminated. Animals began in the dark side and were allowed to freely explore for 10 min with access to the right (light) side through an open doorway located in the center of the monitor. Subject side preference and emergence latencies were tracked by centroid location with all movement automatically tracked and quantified. Light-side emergence latency, time to reach the center of the lighted side (light side center latency) and percent total light-side duration were calculated from beam breaks. All equipment was wiped clean between tests.

#### Contextual and cued fear conditioning

Sound-attenuated isolation cubicles (Coulbourn Instruments, Holliston, MA, USA) were utilized. Each cubicle was equipped with a grid floor for delivery of the unconditioned stimulus (US) and overhead cameras. All aspects of the test were controlled and monitored by the Freeze Frame conditioning and video tracking system (Actimetrics, Coulbourn Instruments). During training the chambers were scented with almond extract, lined with white paper towels, had background noise generated by a small fan and were cleaned before and between trials with 70% ethanol. Each subject was placed inside the conditioning chamber for 2 min before the onset of a conditioned stimulus (CS; an 80 dB, 2 kHz tone), which lasted for 20 s with a co-terminating 2 s footshock (0.7 mA; unconditioned stimulus [US]). A total of three tone/shock pairings were administered separated by one min. Each rat remained in the chamber for an additional 40 s following the last CS-US pairing before being returned to its home cage. Freezing was defined as a lack of movement (except for respiration) in each 10-s interval. Minutes 0–2 during the training session were used to measure baseline freezing. Contextual fear memory testing was performed 24 h after the training session by measuring freezing behavior during a 4-min test in the conditioning chamber under conditions identical to those of the training session with the exception that no footshock or tone (CS or US) was presented. Animals were returned to their home cage for another 24 h at which time cued conditioning was tested. To create a new context with different properties, the chambers were free of background noise (fan turned off), lined with blue paper towels, scented with lemon extract and cleaned before and during all trials with isopropanol. Each subject was placed in this novel context for 2 min and baseline freezing was measured, followed by exposure to the CS (20-s tone) at 120 and 290 s.

#### Novel object recognition

Rats were habituated to the arena (90 cm length × 60 cm width × 40 cm height) for 20 min, 24 h before training. On the training day, two identical objects were placed on opposite ends of the empty arena, and the rat was allowed to freely explore the objects for 7 min. After a 1-h delay, during which the rat was held in its home cage, one of the two familiar objects was replaced with a novel one, and the rat was allowed to freely explore the familiar and novel object for 5 min to assess short-term memory (STM). After a 24-h delay, during which the rat was held in its home cage, one of the two familiar objects was replaced with a novel one different from the ones used during the short-term memory test. The rat was allowed to explore freely the familiar and novel object for 5 min to assess long-term memory (LTM). Raw exploration times for each object were expressed in seconds. Object exploration was defined as sniffing or touching the object with the vibrissae or when the animal’s head was oriented toward the object with the nose placed at a distance of less than 2 cm from the object. All sessions were recorded by video camera (Sentech, Carrollton TX, USA) and analyzed with ANYMAZE software (San Diego Instruments). In addition, offline analysis by an investigator blind to the blast-exposed status of the animals was performed. Objects to be discriminated were of different size, shape and color and were made of plastic or metal material. The objects consisted of a 330 ml soda can, a metal box and a cup and a plastic tube. All objects were cleaned with 70% ethanol between trials. A discrimination index was calculated with the formula: time exploring the novel object minus time exploring the familiar object/total exploration time X 100.

#### Novel object localization

Novel location recognition was used to assess spatial recognition and visual memory using methods previously described [47, 82] that were modified from Yolken et al. [107]. Briefly, 24 h before training, rats were habituated for 20 min to the same empty arena used for the novel object recognition task. The arena was situated in a well-lit room allowing the rats to see distal visual cues. On the training day, two identical objects were placed in specific locations and the rat was allowed to explore freely the objects for 7 min. The test trial was performed after a 1-h delay during which one object was moved to a different location in the arena and the rat was allowed to explore for 5 min. Time spent investigating the objects in their original or novel locations was recorded. During sessions the arena and objects were cleaned before and between trials with 70% ethanol. A discrimination index was calculated as above.

#### Prepulse inhibition and acoustic startle

Startle magnitude and sensory gating were examined in a 40-trial prepulse inhibition assay (San Diego Instruments). Animals were placed in isolation chambers inside closed Plexiglas tubes, each of which was mounted on a platform resting on an accelerometer. Following a 5 min habituation period with 74 dB background white noise, each animal received 40 randomized trials separated by 20–30 s. Trials consisted of 10 each of background readings taken at 74 dB, startle trials with readings following 40 ms 125 dB tones, prepulse inhibition trials where the 125 dB tone was preceded 100 ms earlier by a 20 ms 79 dB tone and control trials consisting of only the 20 ms 79 dB prepulse. On all trials, maximum magnitude of the animal’s startle (or other motion) was automatically recorded in 500 ms windows by an accelerometer. The tubes were rinsed clean between animals. Percent prepulse inhibition was calculated with the formula 100—(startle response on acoustic prepulse plus pulse stimulus trials/pulse stimulus response alone trials) X 100]. The first startle response was compared among groups.

### Regional brain dissection

To dissect the various brain regions the cerebellum was removed and the brain was placed ventral side up and coronal cuts were made through the optic chiasm and anterior commissure. The neocortical tissue surrounding this piece of tissue was defined as the anterior cortex. To obtain the amygdala fraction, the tissue lateral to the hypothalamus between its caudal and rostral border and ventral to the rhinal sulcus on either side was dissected. After dissection of the amygdala the tissue was turned dorsal side up and the hippocampus dissected based on its typical morphology after the cerebral hemispheres were reflected out. The posterior neocortex included the remainder of the cerebral tissue after the removal of the caudate-putamen.

### Western blot analysis

Tissue was homogenized in 0.1 M Tris HCl buffer pH 7.4, containing 0.15 M NaCl, 5 mM EDTA, 1% Triton X100/0.1% SDS and a protease and phosphatase inhibitor cocktail (Halt, Pierce, Rockford IL, USA). Protein concentration was determined with the BCA reagent (Pierce). Protein samples (50 μg) were separated by SDS-PAGE and blotted onto polyvinylidene difluoride (PVDF) membranes (Millipore Corporation, Billerica, MA, USA). Blots were blocked with 50 mM Tris HCl, pH 7.6, 0.15 M NaCl, 0.1% Tween-20 (TBST), 5% nonfat dry milk and probed overnight with the relevant primary antibody diluted in blocking solution. Blots were then incubated for 1.5 h with the appropriate horseradish peroxidase (HRP) conjugated secondary antibody (GE Healthcare Lifesciences, Piscataway, NJ, USA) diluted in blocking solution (1:7500) and bands were visualized by ECL Prime (GE Healthcare Lifesciences) and imaged with an Imager 600 workstation or by exposure to HyBlot CL film (Denville Scientific). The primary antibodies utilized are indicated in Table 1. All blots were sequentially reprobed with anti-tau to determine total tau levels and with anti glyceraldehyde 3-phosphate dehydrogenase (GAPDH) as loading controls. For reprobing, the membranes were stripped with Re-Blot Plus strong stripping solution (Millipore) or ReblotPlus Western blot stripping buffer (ThermoFisher) according to the manufacturer’s instructions. Quantification was performed using Image Quant TL software (GE Healthcare Lifesciences). Phosphorylated tau (p-tau) levels were determined by adding the two major bands seen in Western blot and normalizing to total tau. For total tau quantitation the sum of the major and minor bands seen in Western blot was used. Total tau levels were normalized to GAPDH. All data were normalized relative to control samples.

**Table 1:** Primary antibodies used for Western blotting

Antibody	Specificity	Source	Type	Dilution
AT270	Thr181	ThermoFisher MN1050	Mouse monoclonal	1:600
Phospho-Tau (Thr181)	Thr181	Cell Signaling 12885	Rabbit monoclonal	1:1200
Phospho-Tau (Thr181)	Thr181	ThermoFisher 710561	Rabbit oligoclonal	1:1000
Phospho-Tau (Ser404)	Ser404	ThermoFisher 44-758G	Rabbit polyclonal	1:1000
PHF13	Ser396	Cell Signaling 9632	Mouse monoclonal	1:1000
Phospho-Tau (Thr231)	Thr231	ThermoFisher 710126	Rabbit oligoclonal	1:1000
CP13	Ser202	Dr. Peter Davies	Mouse monoclonal	1:500
AT8	Ser202/Thr205	ThermoFisher MN1020	Mouse monoclonal	1:600
Tau	Total tau	Proteintech 10274-1-AP	Rabbit polyclonal	1:600

### Statistical analysis

Levels of p-tau isoforms and total tau were determined at 40 weeks after blast exposure in previous studies [21]. Tau levels were correlated with behavioral measures previously reported in the same blast-exposed and control (i.e. sham exposed) rats [80]. The time of behavioral testing in individual tasks is shown in Table 2. Data from five blast and five control animals were pooled for analysis. Data sets were tested for normality in GraphPad Prism 8.0 (GraphPad Software) using the D'Agostino-Pearson normality test. Pearson’s product-moment correlation coefficient, Kendall's tau-b, and Spearman's rho were calculated using SPSS v26 (IBM). Figures were prepared using GraphPad Prism 8.0 and Adobe Photoshop.

**Table 2:** Time following blast exposure when behavioral testing was begun

Task	Time (weeks)
Locomotor activity and open field	28
Light/dark emergence	29
Elevated zero maze	30
Novel object recognition	31
Novel location recognition	33
Acoustic startle/PPI	34
Cued and contextual fear conditioning	35

Original data was reported in Perez Garcia et al. [80]

## Results

### Elevated p-tau threonine 181 (Thr181) in the anterior neocortex and right hippocampus correlates with behavioral measures of anxiety in blast-exposed rats

Rats exposed to repetitive low-level blast develop chronic cognitive and PTSD-related behavioral trait that are present for at least 1 year following blast exposure [78, 81]. Previously we found that p-tau was chronically elevated in rats studied at 6 weeks, 40 weeks and 52 weeks following blast exposure [21]. Patterns are summarized in Table 3. With the anti p-tau antibody AT270 directed against phospho-Thr181, p-tau was increased in the right hippocampus but not the left hippocampus in all three cohorts. In the anterior cortex, p-tau was elevated on the right at all three time points while on the left it was increased at 40 weeks and 52 weeks but not at 6 weeks. P-tau was not changed in the amygdala or posterior cortex at any time point. Increased phosphorylation at p-Thr181 was confirmed using two additional anti-p-Thr181 antibodies besides AT270 [21].

**Table 3:** P-tau phosphorylation at Thr181 by brain region

Brain region/time post-blast exposure	Anterior cortex		Hippocampus		Amygdala		Posterior cortex	
Side	Left	Right	Left	Right	Left	Right	Left	Right
Six weeks	NC	↑	NC	↑	ND	ND	ND	ND
40 weeks	↑	↑	NC	↑	NC	NC	NC	NC
52 weeks	↑	↑	NC	↑	ND	ND	ND	ND

↑ = increased; NC = no change; ND = not determined. Phosphorylation at Thr181 was determined by Western blotting using the antibody AT270 in data previously reported [21]

The rats studied at 40 weeks following blast exposure were also previously characterized behaviorally [80] and found to exhibit the chronic cognitive and PTSD-related behavioral traits that are characteristic of this model [81]. Given the distinctive regional patterns and laterality of blast-induced tau phosphorylation we examined whether these patterns could be correlated with specific behavioral traits.

**Fig. 1:**
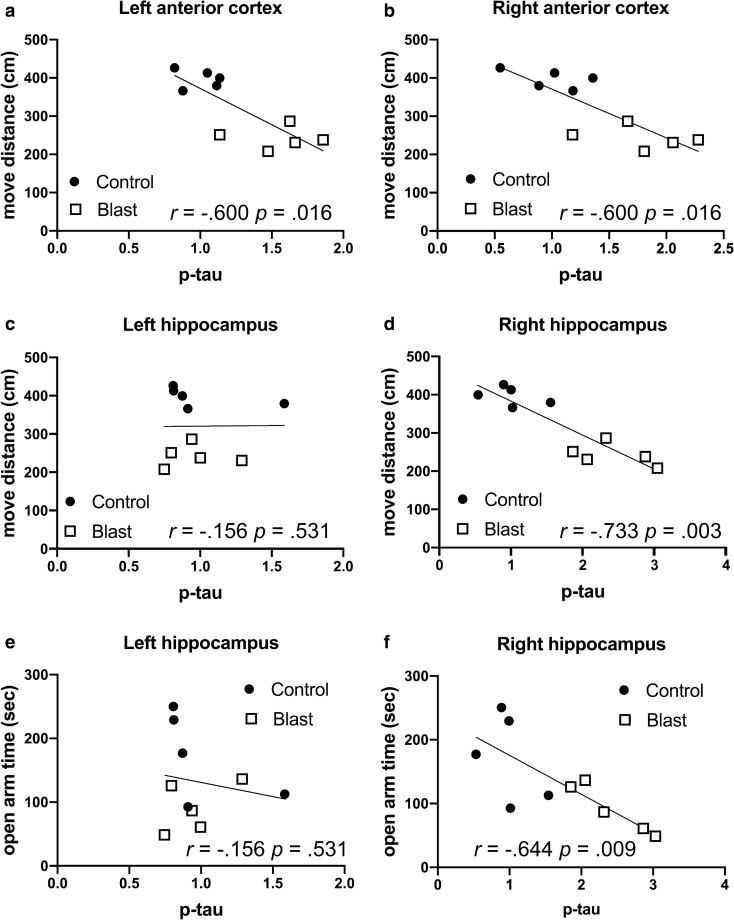
Correlations between p-tau Thr181 in the anterior cortical regions and hippocampus with behavioral measures in the elevated zero maze. P-tau Thr181 levels were previously determined by Western blotting with the antibody AT270 [21] at 40 weeks after blast exposure and correlated with behavioral measures previously found affected in the elevated zero maze in the same blast-exposed rats at 30 weeks following blast exposure [80]. Distance traveled (move distance, **a–d**) and time spent in the open arms (open arm time, **e**, **f**) are shown for the left anterior cortex (**a**), right anterior cortex (**b**), left hippocampus (**c**, **e**) and right hippocampus (**d**, **f**). Five blast and five control animals were analyzed. Kendall's tau-b correlation coefficients and *p* values are indicated

Figure 1 and Table 4 show correlation coefficients between p-Thr181 levels in four brain regions with behavioral measures in the elevated zero maze. In our previously published work, blast-exposed rats exhibited features of anxiety, moving less (move distance) as well as spending less time in the open arms and exhibiting a longer latency to cross between two open arms [80]. Negative correlations were found between p-tau phosphorylation in the left and right anterior cortex, as well as the right hippocampus between move distance (left and right anterior cortex, right hippocampus), open arm time (right anterior cortex, right hippocampus) and cross arm latency (left and right anterior cortex, right hippocampus, Table 4). There were no significant correlations in left hippocampus, left or right amygdala as well as left or right posterior cortex (Table 4).

**Table 4:** Correlations between p-tau Thr181 levels and behavioral parameters in the elevated zero maze

Elevated zero maze						
p-tau (Thr181)	Pearson		Kendall's tau-b		Spearman's rho	
	Correlation coefficient	*p* value	Correlation coefficient	*p* value	Correlation coefficient	*p* value
*Left anterior cortex*						
Move distance	** − .792**	**.006**	** − .600**	**.016**	** − .806**	**.005**
Open arm time	− .620	.056	− .422	.089	− .576	.082
Cross arm latency	.624	.054	**.556**	**.025**	**.782**	**.008**
*Right anterior cortex*						
Move distance	** − .814**	**.004**	** − .600**	**.016**	** − .806**	**.005**
Open arm time	** − .676**	**.032**	** − .511**	**.040**	− .624	.054
Cross arm latency	.493	.148	.467	.060	**.721**	**.019**
*Left Hippocampus*						
Move distance	.010	.979	− .156	.531	− .055	.881
Open arm time	− .173	.633	− .156	.531	− .115	.751
Cross arm latency	− .033	.928	.022	.929	.042	.907
*Right Hippocampus*						
Move distance	** − .901**	** < .001**	** − .733**	**.003**	** − .891**	**.001**
Open arm time	** − .755**	**.008**	** − .644**	**.009**	** − .842**	**.002**
Cross arm latency	.564	.090	**.689**	**.006**	**.879**	**.001**
*Left Amygdala*						
Move distance	.120	.741	.156	.531	.248	.489
Open arm time	.118	.745	− .022	.929	.042	.907
Cross arm latency	.415	.233	− .111	.655	− .103	.777
*Right Amygdala*						
Move distance	− .016	.966	.111	.655	.127	.726
Open arm time	.198	.584	.289	.245	.382	.276
Cross arm latency	− .293	.412	− .244	.325	− .285	.425
*Left Posterior Cortex*						
Move distance	− .074	.839	.067	.788	.103	.777
Open arm time	.057	.877	.156	.531	.055	.881
Cross arm latency	.499	.142	.244	.325	.345	.328
*Right Posterior Cortex*						
Move distance	.199	.581	.111	.655	.224	.533
Open arm time	.479	.161	.200	.421	.345	.328
Cross arm latency	− .105	.772	− .067	.788	− .091	.803

P-tau Thr181 levels were previously determined by Western blotting with the antibody AT270 at 40 weeks after blast exposure [21] and correlated with three behavioral measures in the elevated zero maze (move distance, open arm time and cross arm latency) previously found to be affected in the same blast-exposed rats at 30 weeks following blast exposure [80]. Five blast and five control animals were analyzed. Correlations with *p* values less than 0.05 are indicated in bold

**Fig. 2:**
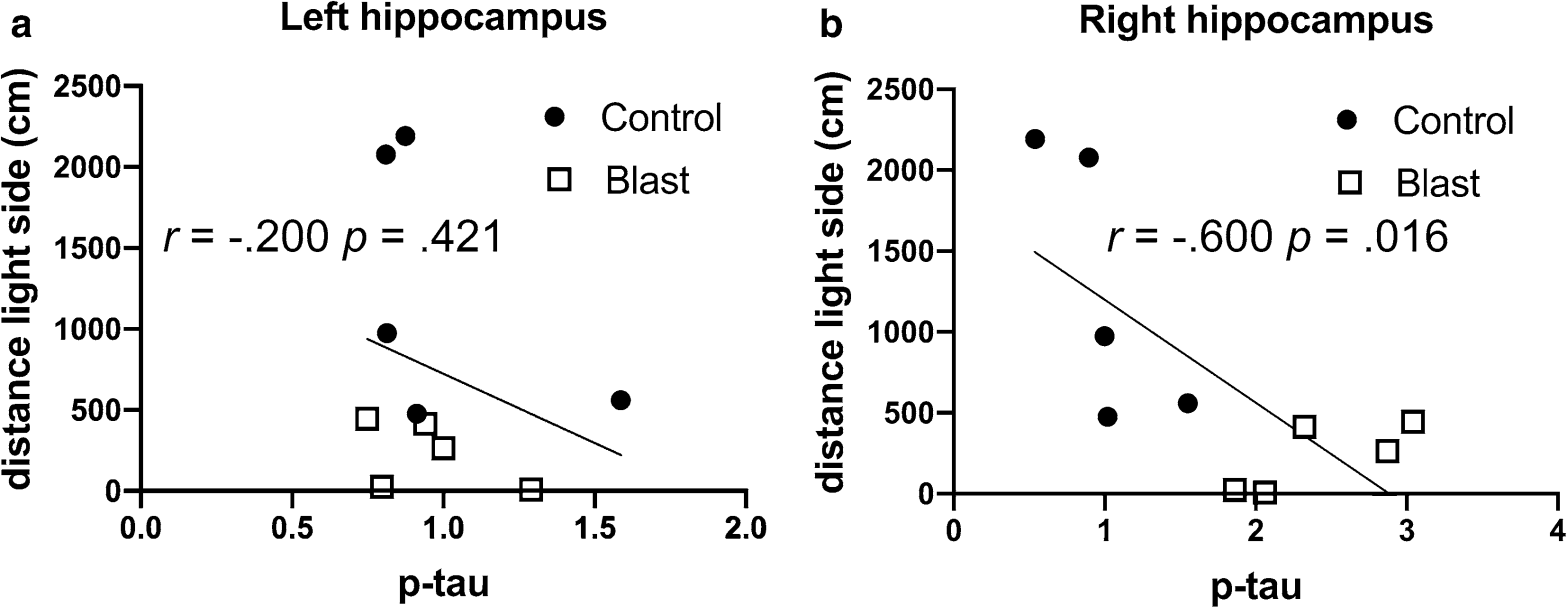
Correlations between p-tau Thr181 levels and behavioral parameters in the light dark escape task. P-tau Thr181 levels [21] in the left (**a**) or right (**b**) hippocampus were determined at 40 weeks after blast exposure and correlated with total distance moved on the light side in the same animals tested at 29 weeks after blast exposure [80]. Five blast and five control animals were analyzed. Kendall's tau-b correlation coefficients and *p* values are indicated

**Fig. 3:**
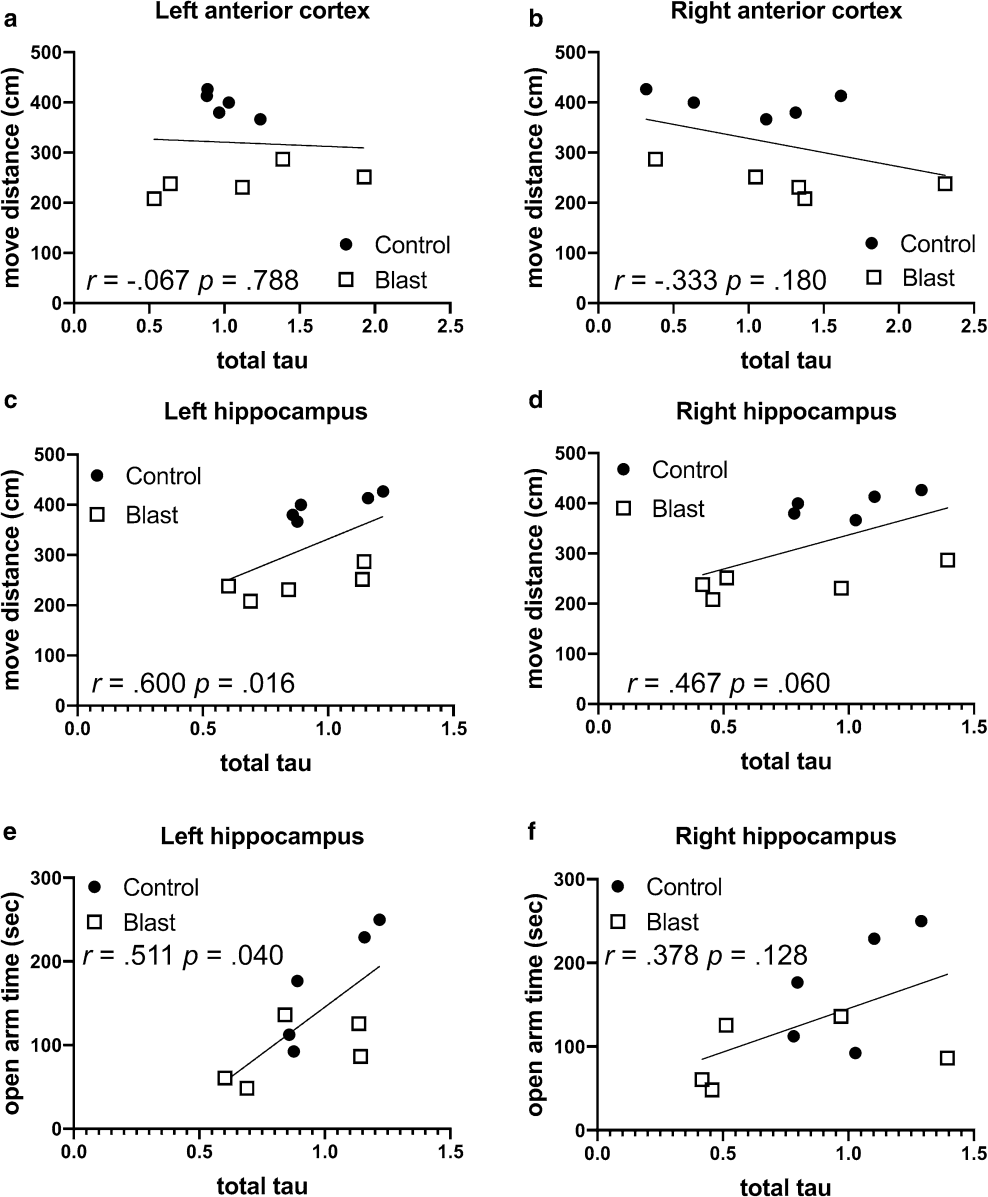
Correlations between total tau levels and behavioral parameters in the elevated zero maze. Total tau levels were previously determined by Western blotting [21] at 40 weeks after blast exposure and correlated with behavioral measures previously found affected in the elevated zero maze in the same blast-exposed rats at 30 weeks following blast exposure [80]. Distance traveled (move distance, **a–d**) and time spent in the open arms (open arm time, **e**, **f**) are shown for the left anterior cortex (**a**), right anterior cortex (**b**), left hippocampus (**c**, **e**) and right hippocampus (**d**, **f**). Five blast and five control animals were analyzed. Kendal's tau-b correlation coefficients and *p* values are indicated

Similar correlations were seen in the left anterior cortex and right hippocampus in time spent and distance traveled on the light side of a light/dark escape task (Fig. 2 and Table 5), another test of anxiety, which is consistently affected in blast-exposed rats [81]. Changes were specific to p-tau in that there were no consistent correlations between behavior in the elevated zero maze and total tau in regions where p-tau Thr181 correlated with behavior (Fig. 3 and Table 6). The only significant correlations were positive correlations between total tau in the left hippocampus with move distance and open arm time (Fig. 3c, e and Table 6).

**Table 5:** Correlations between p-tau Thr181 levels and behavioral parameters in the light dark escape task

Light dark escape						
*p-tau (Thr181)*	Pearson		Kendall's tau-b		Spearman's rho	
	Correlation coefficient	*p* value	Correlation coefficient	*p* value	Correlation coefficient	*p* value
*Left anterior cortex*						
Total time light side	− .526	.119	** − .511**	**.040**	** − .673**	**.033**
Total distance light side	− ..545	.103	** − .556**	**.025**	** − .745**	**.013**
*Right anterior cortex*						
Total time light side	− .474	.166	− .333	.180	− .467	.174
Total distance light side	− .544	.104	− .467	.060	− .612	.060
*Left Hippocampus*						
Total time light side	− .447	.195	− .333	.180	− .430	.214
Total distance light side	− .287	.421	− .200	.421	− .212	.556
*Right Hippocampus*						
Total time light side	** − .633**	**.050**	** − .556**	**.025**	** − .648**	**.043**
Total distance light side	** − .700**	**.024**	** − .600**	**.016**	** − .770**	**.009**
*Left Amygdala*						
Total time light side	− .024	.947	− .022	.929	− .091	.803
Total distance light side	.219	.543	.022	.929	− .018	.960
*Right Amygdala*						
Total time light side	− .026	.944	− .067	.788	− .139	.701
Total distance light side	.144	.691	.067	.788	− .018	.960
*Left Posterior Cortex*						
Total time light side	.281	.432	.156	.531	.321	.365
Total distance light side	.067	.853	.111	.655	.176	.627
*Right Posterior Cortex*						
Total time light side	.237	.510	.111	.655	.164	.651
Total distance light side	.406	.244	.156	.531	.212	.556

P-tau Thr181 were measured for the same rats studied in Table 3 at 40 weeks after blast exposure [21] and correlated with total time on the light side or total distance moved on the light side in a light dark escape task tested at 29 weeks after blast exposure [80]. Five blast and five control animals were analyzed. Correlations with *p* values equal to or less than 0.05 are indicated in bold

**Table 6:** Correlations between total tau levels and behavioral parameters in the elevated zero maze

Elevated zero maze						
*Total tau*	Pearson		Kendall's tau-b		Spearman's rho	
	Correlation coefficient	*p* value	Correlation coefficient	*p* value	Correlation coefficient	*p* value
*Left anterior cortex*						
Move distance	− .057	.875	− .067	.788	.018	.960
Open arm time	.040	.913	.022	.929	.127	.726
Cross arm latency	.230	.522	.022	.929	.055	.881
*Right anterior cortex*						
Move distance	− .396	.257	− .333	.180	− .455	.187
Open arm time	− .390	.265	− .244	.325	− .370	.293
Cross arm latency	− .114	.753	.289	.245	.358	.310
*Left Hippocampus*						
Move distance	.508	.134	**.600**	**.016**	**.770**	**.009**
Open arm time	**.696**	**.025**	**.511**	**.040**	**.697**	**.025**
Cross arm latency	.076	.834	− .289	.245	− .394	.260
*Right Hippocampus*						
Move distance	.550	.100	.467	.060	.588	.074
Open arm time	.526	.119	.378	.128	.503	.138
Cross arm latency	.261	.467	− .156	.531	− .152	.676
*Left Amygdala*						
Move distance	.158	.662	.022	.929	.018	.960
Open arm time	.189	.601	.111	.655	.152	.676
Cross arm latency	− .559	.093	− .244	.325	− .297	.405
*Right Amygdala*						
Move distance	.278	.437	.244	.325	.382	.276
Open arm time	− .048	.894	− .022	.929	.018	.960
Cross arm latency	.361	.306	− .111	.655	− .115	.751
*Left Posterior Cortex*						
Move distance	.361	.305	.333	.180	.345	.328
Open arm time	.080	.826	.067	.788	.127	.726
Cross arm latency	− .272	.448	− .378	.128	− .455	.187
*Right Posterior Cortex*						
Move distance	− .629	.051	** − .511**	**.040**	** − .697**	**.025**
Open arm time	− .489	.151	− .244	.325	− .382	.276
Cross arm latency	− .126	.729	.200	.421	.236	.511

Total tau levels were previously determined by Western blotting at 40 weeks after blast exposure [21]. Total tau levels were correlated with three behavioral parameters in the elevated zero maze (move distance, open arm time and cross arm latency) previously found to be affected in blast-exposed animals at 30 weeks following blast exposure [80]. Five blast and five control animals were analyzed. Correlations with *p* values less than 0.05 are indicated in bold

**Fig. 4:**
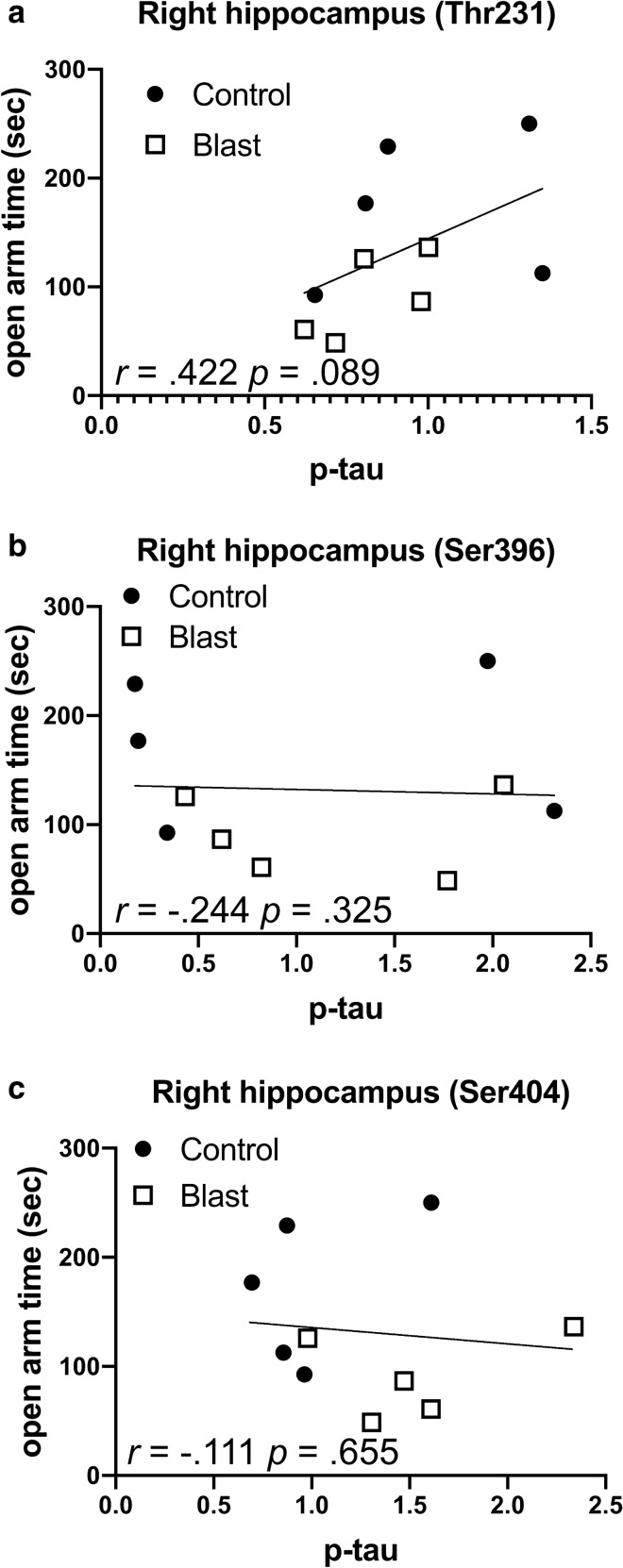
Correlations between levels of p-tau Thr231, Ser396 and Ser404 with open arm time in the elevated zero maze. Levels of p-tau Thr231 (**a**), Ser396 (**b**) and Ser404 (**c**) in the right hippocampus were previously determined by Western blotting [21] at 40 weeks after blast exposure and correlated with open arm time in the elevated zero maze determined at 30 weeks following blast exposure [80]. Five blast and five control animals were analyzed. Kendal's tau-b correlation coefficients and *p* values are indicated

Effects were specific to p-tau Thr181 in that no consistent correlations existed between behavioral parameters in the elevated zero maze (Fig. 4, Tables 7, 8, 9) or light/dark escape task (data not shown) with phosphorylation status at three other sites (threonine 231, serine 396, and serine 404) whose phosphorylation status was not changed following blast [21].

**Table 7:** Correlations between p-tau Thr231 levels and behavioral parameters in the elevated zero maze

Elevated zero maze						
*p-tau Thr231*	Pearson		Kendall's tau-b		Spearman's rho	
	Correlation coefficient	*p* value	Correlation coefficient	*p* value	Correlation coefficient	*p* value
*Left anterior cortex*						
Move distance	− .373	.288	− .244	.325	− .309	.385
Open arm time	− .540	.107	− .333	.180	− .455	.187
Cross arm latency	.087	.811	.022	.929	.018	.960
*Right anterior cortex*						
Move distance	.139	.702	− .067	.788	− .139	.701
Open arm time	− .146	.687	− .156	.531	− .297	.405
Cross arm latency	− .059	.871	.022	.929	− .006	.987
*Left Hippocampus*						
Move distance	.057	.876	.111	.655	.139	.701
Open arm time	− .305	.392	− .244	.325	− .285	.425
Cross arm latency	.396	.257	.022	.929	.055	.881
*Right Hippocampus*						
Move distance	.455	.186	.333	.180	.442	.200
Open arm time	.489	.151	.422	.089	.552	.098
Cross arm latency	− .158	.662	− .200	.421	− .285	.425

P-tau Thr231 levels were previously determined by Western blotting at 40 weeks after blast exposure [21]. P-tau Thr231 levels were correlated with three behavioral parameters in the elevated zero maze (move distance, open arm time and cross arm latency) determined at 30 weeks following blast exposure [80]. Five blast and five control animals were analyzed. No values were statistically significant

**Table 8:** Correlations between p-tau Ser396 levels and behavioral parameters in the elevated zero maze

Elevated zero maze						
p-tau Ser396	Pearson		Kendall's tau-b		Spearman's rho	
	Correlation coefficient	*p* value	Correlation coefficient	*p* value	Correlation coefficient	*p* value
*Left anterior cortex*						
Move distance	− .632	.050	− .467	.060	− .612	.060
Open arm time	− .473	.167	− .378	.128	− .491	.150
Cross arm latency	.125	.732	.333	.180	.418	.229
*Right anterior cortex*						
Move distance	− .563	.090	− .422	.089	** − .636**	**.048**
Open arm time	− .331	.350	− .333	.180	− .455	.187
Cross arm latency	.229	.524	.289	.245	.491	.150
*Left Hippocampus*						
Move distance	− .360	.307	− .244	.325	− .382	.276
Open arm time	− .314	.377	− .244	.325	− .333	.347
Cross arm latency	.535	.111	.289	.245	.442	.200
*Right Hippocampus*						
Move distance	− .157	.665	− .333	.180	− .309	.385
Open arm time	− .051	.888	− .244	.325	− .176	.627
Cross arm latency	− .165	.649	.111	.655	.091	.803

P-tau Ser396 levels were previously determined by Western blotting at 40 weeks after blast exposure [21]. P-tau Ser396 levels were correlated with three behavioral parameters in the elevated zero maze (move distance, open arm time and cross arm latency) determined at 30 weeks following blast exposure [80]. Five blast and five control animals were analyzed. Correlations with *p* values less than 0.05 are indicated in bold

**Table 9:** Correlations between p-tau Ser404 levels and behavioral parameters in the elevated zero maze

Elevated zero maze						
p-tau Ser404	Pearson		Kendall's tau-b		Spearman's rho	
	Correlation coefficient	*p* value	Correlation coefficient	*p* value	Correlation coefficient	*p* value
*Left anterior cortex*						
Move distance	− .482	.158	− .244	.325	− .382	.276
Open arm time	− .219	.543	− .067	.788	− .176	.627
Cross arm latency	.468	.172	.467	.060	**.648**	**.043**
*Right anterior cortex*						
Move distance	− .495	.146	− .378	.128	− .539	.108
Open arm time	− .481	.159	− .378	.128	− .552	.098
Cross arm latency	.219	.544	.333	.180	.564	.090
*Left Hippocampus*						
Move distance	− .309	.385	− .244	.325	− .358	.310
Open arm time	− .280	.434	− .156	.531	− .248	.489
Cross arm latency	.299	.402	.111	.655	.285	.425
*Right Hippocampus*						
Move distance	− .520	.123	− .378	.128	− .418	.229
Open arm time	− .110	.762	− .111	.655	− .103	.777
Cross arm latency	.294	.409	.422	.089	.430	.214

P-tau Ser404 levels were previously determined by Western blotting at 40 weeks after blast exposure [21]. P-tau Ser404 levels were correlated with three behavioral parameters in the elevated zero maze (move distance, open arm time and cross arm latency) determined at 30 weeks following blast exposure [80]. Five blast and five control animals were analyzed. Correlations with *p* values less than 0.05 are indicated in bold

### P-Thr181 levels in the anterior neocortical regions and right hippocampus correlate with behavioral measures of fear learning and novel object localization in blast-exposed rats

**Fig. 5:**
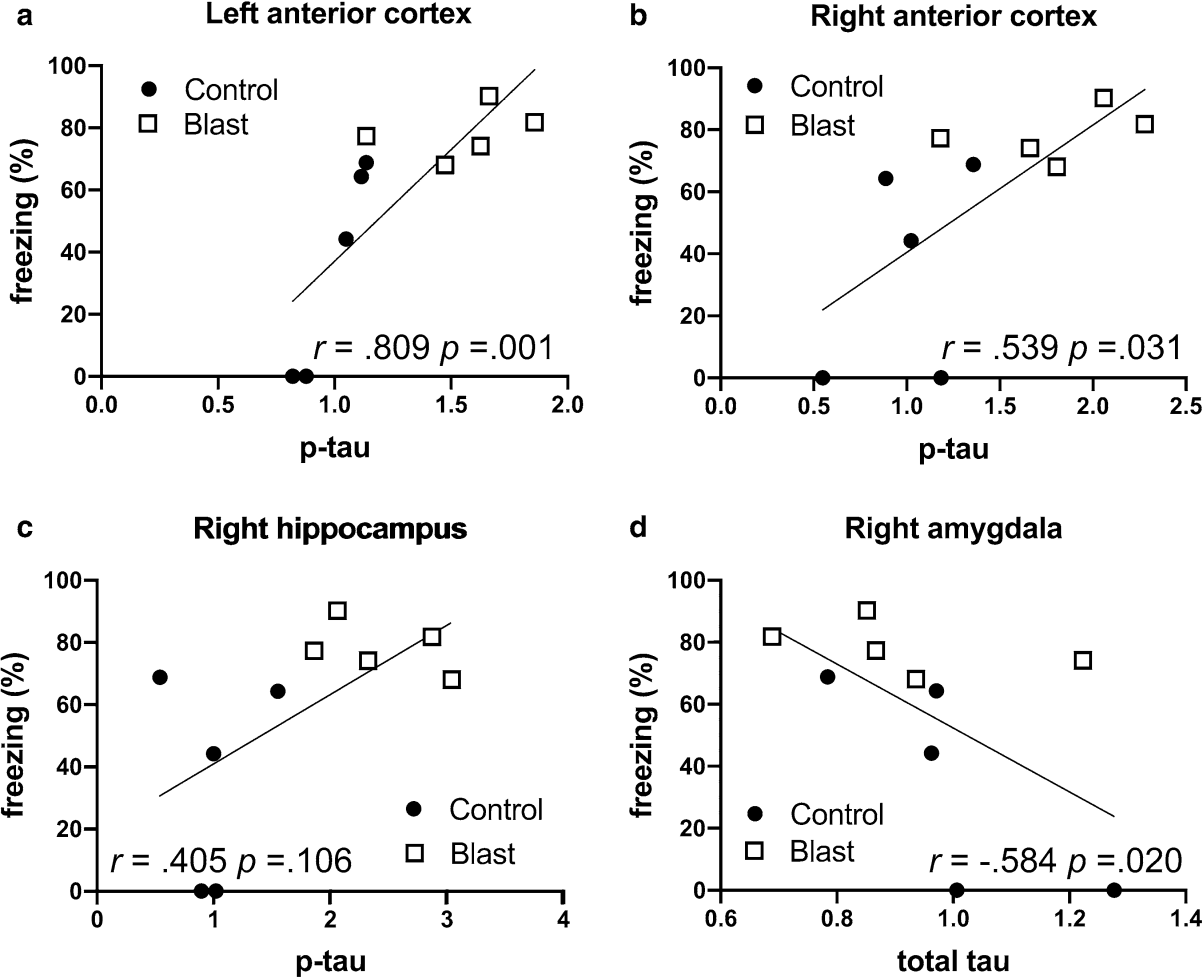
Correlations between p-tau Thr181 levels with freezing in the cued phase of a fear-conditioning paradigm. P-tau Thr181 levels were previously determined by Western blotting with the antibody AT270 [21] in the left anterior cortex (**a**), right anterior cortex (**b**), and right hippocampus (**c**) at 40 weeks after blast exposure. Levels were correlated with freezing to the second tone in the cued testing phase of a fear learning task previously found to be affected in the same blast-exposed rats at 35 weeks following blast exposure [80]. Panel **d** presents the correlation between total tau in the right amygdala and freezing. Five blast and five control animals were analyzed. Kendal's tau-b correlation coefficients and *p* values are indicated

Besides anxiety blast-exposed rats exhibit a variety of other PTSD-related behavioral traits [78, 81]. Abnormal fear responses are a core feature of human PTSD and the rats under study here exhibited normal contextual fear learning but exaggerated cued responses suggestive of heightened amygdala activity [80]. There were no correlations between p-tau or total tau levels and total freezing during the 4 min of the contextual testing (data not shown), consistent with there being no differences between blast and control in this phase of the task as previously determined in these same animals [80]. By contrast in the cued phase comparing p-tau levels with freezing to the second tone showed positive correlations between p-tau Thr181 levels and magnitude of freezing in the left and right anterior cortex (Fig. 5a, b and Table 10) but not the right hippocampus (Fig. 5c). When total tau levels were compared, only a single negative correlation was found between total tau and freezing in the right amygdala (Fig. 5d and Table 10).

**Table 10:** Correlations between p-tau Thr181 and total tau levels with freezing in the cued phase of a fear-conditioning paradigm

Cued Fear (freezing)						
	Pearson		Kendall's tau-b		Spearman's rho	
*p-tau (Thr181) vs. freezing*	Correlation coefficient	*p* value	Correlation coefficient	*p* value	Correlation coefficient	*p* value
Left anterior cortex	**.789**	**.007**	**.809**	**.001**	**.924**	** < 0.001**
Right anterior cortex	**.692**	**.031**	**.539**	**.031**	**.736**	**.015**
Left Hippocampus	.296	.406	.270	.281	.304	.393
Right Hippocampus	.593	.071	.405	.106	.565	.089
Left Amygdala	− .052	.887	.090	.719	.116	.751
Right Amygdala	.323	.363	.315	.209	.438	.206
Left Posterior Cortex	.095	.795	.000	1.000	− .018	.960
Right Posterior Cortex	− .211	.553	.045	.857	− .030	.934
**Total tau vs. freezing**						
Left anterior cortex	.085	.816	.180	.472	.201	.578
Right anterior cortex	.347	.326	.180	.472	.261	.466
Left Hippocampus	− .376	.284	− .360	.151	− .486	.154
Right Hippocampus	− .484	.157	− .315	.209	− .432	.213
Left Amygdala	− .017	.963	− .135	.590	− .243	.498
Right Amygdala	− .581	.078	** − .584**	**.020**	** − .711**	**.021**
Left Posterior Cortex	− .243	.500	− .180	.472	− .316	.374
Right Posterior Cortex	.393	.262	.360	.151	.523	.121

P-tau Thr181 and total tau levels were previously determined by Western blotting with the antibody AT270 at 40 weeks after blast exposure [21]. P-tau levels were correlated with freezing to the second tone in the cued testing phase of a fear learning task previously found to be affected in the same blast-exposed rats at 35 weeks following blast exposure [80]. Five blast and five control animals were analyzed. Correlations with *p* values less than 0.05 are indicated in bold

Mild cognitive changes are a consistent feature of human PTSD [103]. The blast-exposed rats studied here previously showed normal recognition learning in a novel object recognition (NOR) task [78, 80]. In comparing p-tau levels to the NOR discrimination index (Table 11), a measure of the tendency of the animal to prefer the novel over the familiar object when recognition memory is tested later, there was only one positive correlation with p-tau Thr181 in the posterior cortex during the short-term memory (STM) testing conducted 2 h after the training session. However, this correlation was only found in Kendall's tau b, and was not replicated in Pearson's correlation coefficient or Spearman's rho (Table 11). In long-term memory (LTM) testing conducted 24 h after the training session, no correlations were found with p-tau in any region (data not shown). There were also no correlations between total tau and the discrimination index in either STM (Table 11) or LTM testing (data not shown) in the NOR. The paucity of correlations is consistent with there being no impairment in NOR learning in this cohort of blast-exposed rats [80].

**Table 11:** Correlations between p-tau Thr181 and total tau levels with the discrimination index in a novel object recognition task

Novel object recognition (short-term memory)						
*p-tau (Thr181) vs. discrimination index*	Pearson		Kendall's tau-b		Spearman's rho	
	Correlation coefficient	*p* value	Correlation coefficient	*p* value	Correlation coefficient	*p* value
Left anterior cortex	− .132	.716	− .156	.531	− .176	.627
Right anterior cortex	− .373	.289	− .244	.325	− .382	.276
Left Hippocampus	.049	.893	− .067	.788	− .055	.881
Right Hippocampus	− .026	.943	.067	.788	− .006	.987
Left Amygdala	− .015	.967	− .022	.929	− .224	.533
Right Amygdala	− .149	.680	− .156	.531	− .176	.627
Left Posterior Cortex	.545	.104	**.511**	**.040**	.527	.117
Right Posterior Cortex	.035	.924	.200	.421	.297	.405
**Total tau vs. discrimination index**						
Left anterior cortex	− .330	.352	− .333	.180	− .491	.150
Right anterior cortex	− .137	.706	.022	.929	.103	.777
Left Hippocampus	.205	.570	.156	.531	.200	.580
Right Hippocampus	.052	.886	.022	.929	.079	.829
Left Amygdala	.337	.340	.289	.245	.382	.276
Right Amygdala	− .042	.908	.156	.531	.176	.627
Left Posterior Cortex	.227	.529	− .111	.655	− .091	.803
Right Posterior Cortex	− .364	.300	− .333	.180	− .479	.162

P-tau Thr181 levels were previously determined by Western blotting with the antibody AT270 at 40 weeks after blast exposure [21]. P-tau levels were correlated with the discrimination index in a novel object recognition task previously found to be affected in the same blast-exposed rats at 33 weeks following blast exposure [80]. Five blast and five control animals were analyzed. Correlations with *p* values less than 0.05 are indicated in bold

**Fig. 6:**
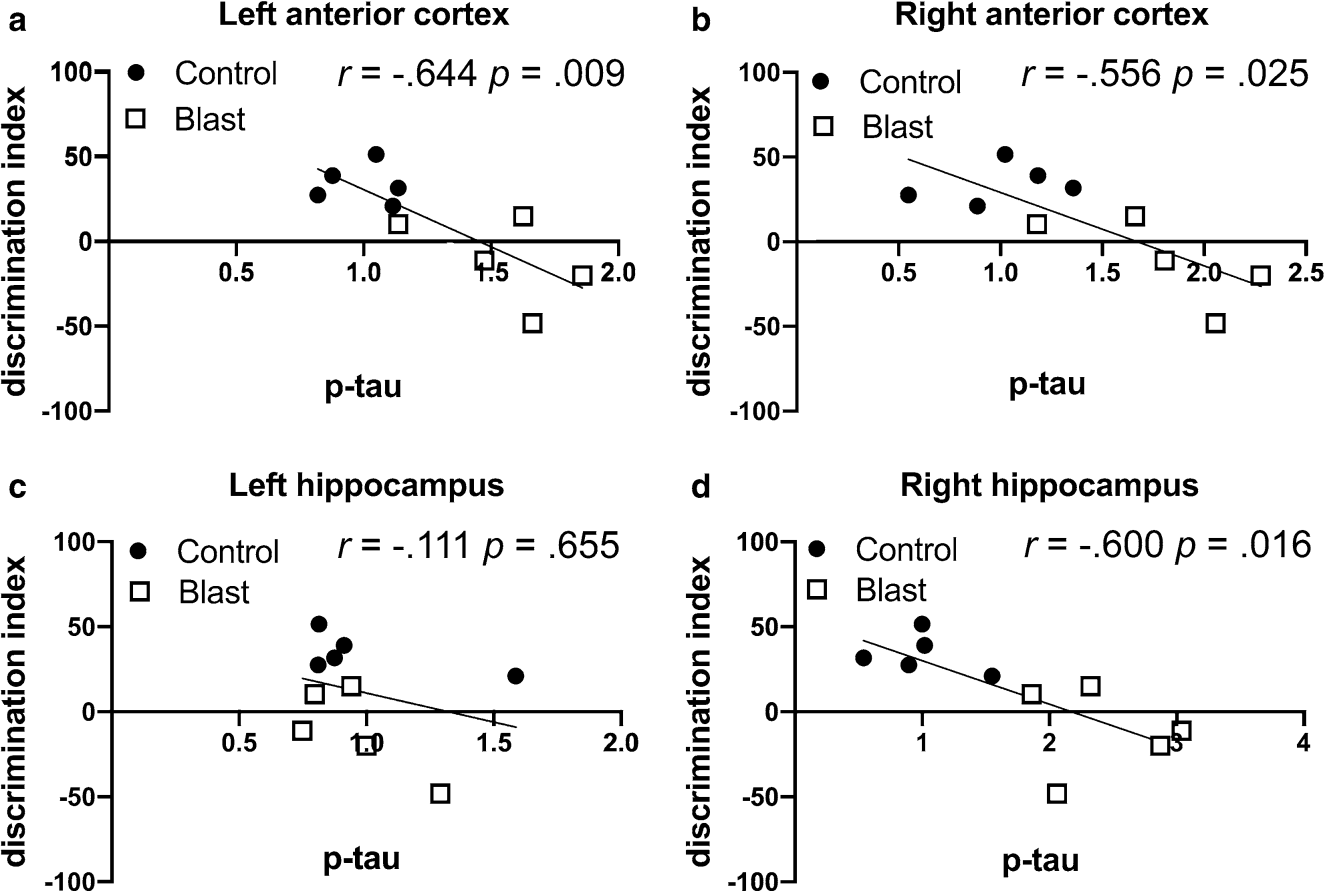
Correlations between p-tau Thr181 levels with the discrimination index in a novel object localization task. P-tau Thr181 levels were previously determined by Western blotting with the antibody AT270 [21] in the left anterior cortex (**a**), right anterior cortex (**b**), left hippocampus (**c**) and right hippocampus (**d**) at 40 weeks after blast exposure. Levels were correlated with the discrimination index in a novel object localization task previously found affected in the same blast-exposed rats at 33 weeks following exposure [80]. Five blast and five control animals were analyzed. Kendal's tau-b correlation coefficients and *p* values are indicated

By contrast these same blast-exposed rats were impaired in recognition memory in a novel object localization (NOL) task [80]. In NOL testing there were negative correlations in left anterior cortex, right anterior cortex and right hippocampus between p-tau Thr181 levels and the discrimination index (Fig. 6 and Table 12). There were only isolated positive or negative correlations of total tau with the discrimination index in the left hippocampus and right posterior cortex that were not replicated across all three correlation coefficients (Table 12).

**Table 12:** Correlations between p-tau Thr181 and total tau levels with the discrimination index in a novel object localization task

Novel object localization (discrimination index)						
*p-tau (Thr181)*	Pearson		Kendall's tau-b		Spearman's rho	
	Correlation coefficient	*p* value	Correlation coefficient	*p* value	Correlation coefficient	*p* value
Left anterior cortex	** − .791**	**.006**	** − .644**	**.009**	** − .842**	**.002**
Right anterior cortex	** − .781**	**.008**	** − .556**	**.025**	** − .685**	**.029**
Left Hippocampus	− .299	.402	− .111	.655	− .200	.580
Right Hippocampus	** − .732**	**.016**	** − .600**	**.016**	** − .794**	**.006**
Left Amygdala	.110	.762	− .067	.788	− .030	.934
Right Amygdala	− .377	.283	− .111	.655	− .273	.446
Left Posterior Cortex	.136	.707	.022	.929	.055	.881
Right Posterior Cortex	− .238	.509	− .111	.655	− .127	.726
**Total tau vs. discrimination index**						
Left anterior cortex	.142	.696	− .022	.929	.055	.881
Right anterior cortex	− .367	.296	.200	.421	− .285	.425
Left Hippocampus	.562	.091	.467	.060	**.648**	**.043**
Right Hippocampus	.409	.240	.422	.089	.527	.117
Left Amygdala	− .142	.695	− .111	.655	− .111	− .067
Right Amygdala	.462	.179	.378	.128	.515	.128
Left Posterior Cortex	.151	.677	.111	.655	.127	.726
Right Posterior Cortex	** − .704**	**.023**	− .467	.060	** − .673**	**.033**

P-tau Thr181 levels were previously determined by Western blotting with the antibody AT270 at 40 weeks after blast exposure. [21]. P-tau levels were correlated with the discrimination index in a novel object localization task previously found to be affected in the same blast-exposed rats at 33 weeks following blast exposure [80]. Five blast and five control animals were analyzed. Correlations with *p* values less than 0.05 are indicated in bold

### P-tau Thr 181 levels do not correlate with behavioral measures of hyperarousal in blast-exposed rats

Hyperarousal is a core feature of PTSD [103] and acoustic startle response is increased in humans with PTSD [12, 72, 77]. When control and blast-exposed animals were previously examined in an acoustic startle/prepulse inhibition (PPI) paradigm, while there were no overall group differences in response to acoustic startle, blast-exposed rats exhibited an increased percentage PPI and showed a larger differential when the response following a prepulse was subtracted from the acoustic startle [80]. However, no correlations were found between p-tau Thr181 levels and the percentage PPI or when the prepulse response was subtracted from the acoustic startle (Table 13).

**Table 13:** Correlations between p-tau Thr181 levels and parameters in acoustic startle and prepulse inhibition

Acoustic startle/prepulse inhibition						
p-tau (Thr181)	Pearson		Kendall's tau-b		Spearman's rho	
	Correlation coefficient	*p* value	Correlation coefficient	*p* value	Correlation coefficient	*p* value
*Left anterior cortex*						
Pulse-prepulse	− .245	.496	− .111	.655	− .067	.855
PPI (%)	.317	.371	.289	.245	.467	.174
*Right anterior cortex*						
Pulse-prepulse	− .221	.539	− .111	.655	− .042	.907
PPI (%)	.314	.376	.289	.245	.358	.310
*Left Hippocampus*						
Pulse-prepulse	− .520	.123	− .378	.128	− .503	.138
PPI (%)	− .047	.898	− .244	.325	− .333	.347
*Right Hippocampus*						
Pulse-prepulse	− .179	.620	− .156	.531	− .224	.533
PPI (%)	.430	.215	.244	.215	.418	.229
*Left Amygdala*						
Pulse-prepulse	.506	.136	.289	.245	.479	.162
PPI (%)	.146	.688	.244	.325	.164	.651
*Right Amygdala*						
Pulse-prepulse	− .240	.505	− .022	.929	− .127	.726
PPI (%)	− .105	.774	− .067	.788	− .091	.803
*Left Posterior Cortex*						
Pulse-prepulse	− .080	.825	− .067	.788	− .067	.855
PPI (%)	− .247	.492	− .022	.929	− .018	.960
*Right Posterior Cortex*						
Pulse-prepulse	.155	.669	.067	.788	.067	.855
PPI (%)	.151	.677	.111	.655	.127	.726

P-tau Thr181 levels were previously determined by Western blotting with the antibody AT270 at 40 weeks after blast exposure [21] and correlated with measures previously found to be affected in the same blast-exposed rats at 34 weeks following blast exposure [80]. Pulse refers to the first startle reading. Pulse-prepulse refers to background readings (prepulse) subtracted from the startle (pulse). Percent prepulse inhibition (PPI) was calculated with the formula 100 − (startle response on acoustic prepulse plus pulse stimulus trials/pulse stimulus response alone trials) × 100]. Five blast and five control animals were analyzed. There were no statistically significant correlations

## Discussion

While symptoms following mTBI frequently resolve, many veterans’ complaints persist and evolve into a chronic postconcussion syndrome that can last for years. Besides static symptoms, new complaints may also develop [53, 64]. In a longitudinal study of US military personnel who suffered blast-related TBIs in Afghanistan, with 1 and 5 years of follow-up times, overall global functioning declined in over 70%, declines nearly completely driven by worsening PTSD and depression [64]. The degree to which this worsening is driven by blast-related mechanisms vs. purely mental health-related factors is unclear.

This study utilized an animal model that was designed to mimic a blast-related human mild TBI or subclinical blast exposure. Studies using this system established that exposures up to 74.5 kPa (equivalent to 10.8 psi), while representing a level of blast that is transmitted to brain [16] produce no gross neuropathological effects, and histological examination of the lung shows no hemorrhage or other pathology [1]. Because blast-related TBI may involve a combination of injuries related to effects of the primary blast wave as well as damage from rotational/acceleration injury [15, 92], during the blast overpressure exposures head motion is restricted to minimize rotation/acceleration injury. The lack of evidence for coup/contrecoup injuries or brain tissue damage generally on histology supports the mild nature of the injury and the lack of significant rotation/acceleration injury [1, 26]. Because multiple blast exposures were common among veterans returning from Iraq and Afghanistan [28], in most experiments (including the present) we have used a design in which rats received three 74.5-kPa exposures delivered one exposure per day on 3 consecutive days.

Altered tau processing has frequently been observed in experimental animal models of blast injury [2, 4, 5, 11, 13, 17, 22, 23, 35, 39, 43, 44, 52, 57, 58, 61–63, 67, 70, 73, 83, 86, 88]. The blast-exposed rats studied here develop a variety of chronic and persistent PTSD-related behavioral traits [81] following repetitive low-level blast exposure. They also accumulate abnormal levels of p-tau in a region-specific pattern [21].

Here, we compared region-specific patterns of tau phosphorylation with previous behavioral testing performed on the same rats [80] using data sets that were published in prior studies [21, 80]. The results are summarized in Table 14. The most consistent correlations were with p-tau levels in the left anterior cortex, right anterior cortex and right hippocampus, regions in which p-tau was elevated (Table 3). In these regions, p-tau levels correlated negatively with measures of anxiety in an elevated zero maze and a light/dark escape task as well as object localization memory in an NOL task while they correlated positively with freezing in the cued phase of a fear-conditioning paradigm. All these correlations are consistent with evidence of anxiety, impaired NOL learning and exaggerated fear responses in blast-exposed rats [80]. Only isolated positive or negative correlations were found between total tau in the left hippocampus and measures of anxiety or fear learning (Table 14).

**Table 14:** Summary of p-tau Thr181 and total tau correlations across brain regions with behavioral tests

p-tau Thr181	Left anterior cortex	Right anterior cortex	Left hippocampus	Right hippocampus	Left amygdala	Right amygdala
EZM/LD	↓	↓	NC	↓	NC	NC
Fear conditioning	↑	↑	NC	NC	NC	NC
NOL	↓	↓	NC	↓	NC	NC
Acoustic startle/PPI	NC	NC	NC	NC	NC	NC
**Total tau**						
EZM/LD	NC	NC	↑	NC	NC	NC
Fear conditioning	NC	NC	NC	NC	NC	↓
NOL	NC	NC	NC	NC	NC	NC
Acoustic startle/PPI	NC	NC	NC	NC	NC	NC

↑ = positive correlation; ↓ = negative correlation; NC = no correlation; EZM = elevated zero maze; LD = light/dark escape task; NOL = novel object localization; PPI = prepulse inhibition

A limitation of these studies is the relatively modest sample size of animals for which both behavioral and quantitative tau phosphorylation data were available. There are also challenges with statistical testing of relatively small sample sizes. While most data sets did not violate normality (data not shown) we calculated both parametric (Pearson’s product moment) and non-parametric (Kendall's tau-b and Spearman's rho) correlation tests. Agreement between the three tests was generally high with only a few instances in which Kendall's tau-b and/or Spearman's rho yielded significant correlations while Pearson’s did not. Kendall's tau-b is generally considered to give better estimates of the population value with small data sets or when data has failed one or more assumptions [32, 42]. For those reasons Kendall's tau-b is presented in figures. It remains possible that an increased sample size would identify more statistically significant correlations.

The rat model under study here also develops pathological accumulation of hyperphosphorylated tau in neuronal perikarya and perivascular astroglial processes in multiple brain regions [21]. In future studies it will be interesting to explore the relationship of these accumulations with behavioral measures as well as whether the degenerative pathology spreads in a manner comparable to that observed in a variety of human neurodegenerative diseases [24]. Several groups including our own have observed retention of the PET tau ligand [^18^F]AV1451 (flortaucipir) in military veterans who suffered from chronic cognitive and neurobehavioral syndromes after blast injury [21, 71, 84]. It may be possible to better understand the laterality and spread of disease using similar ligands in rats.

These studies also suggest tau accumulations as a possible therapeutic target following blast injury. Studies in mice have suggested that tau KO mice are resistant to stress-induced behavioral changes and hippocampal pathology [60]. Whether knocking down tau on a regional basis could reverse blast-induced behavioral changes could be tested in rats. The microtubule stabilizing agent epothilone D has been shown to reduce pathological aggregation and phosphorylation of tau and lead to improvements in behavioral function in transgenic mouse models of human tauopathies [7, 10, 109]. It would of great translational relevance to test whether microtubule stabilizing agents such epothilone D can reverse the chronic behavioral effects that follow blast injury in rats.

Current biological models of PTSD hold that specific brain regions, including the prefrontal cortex, amygdala, and hippocampus, are involved in its development [55, 56, 66]. These models suggest that a key component of the disorder is inadequate frontal inhibition of the amygdala, a structure central to fear responses and formation of fear associations [55, 56]. Exaggerated amygdala responses are thought to heighten responses to psychological threats. Functional neuroimaging data in humans are consistent with such models, suggesting that heightened amygdala activity is associated with decreased hippocampal and orbitofrontal activity in PTSD [56]. These areas strongly overlap with brain areas known to support fear learning in rodents [81, 100].

**Fig. 7:**
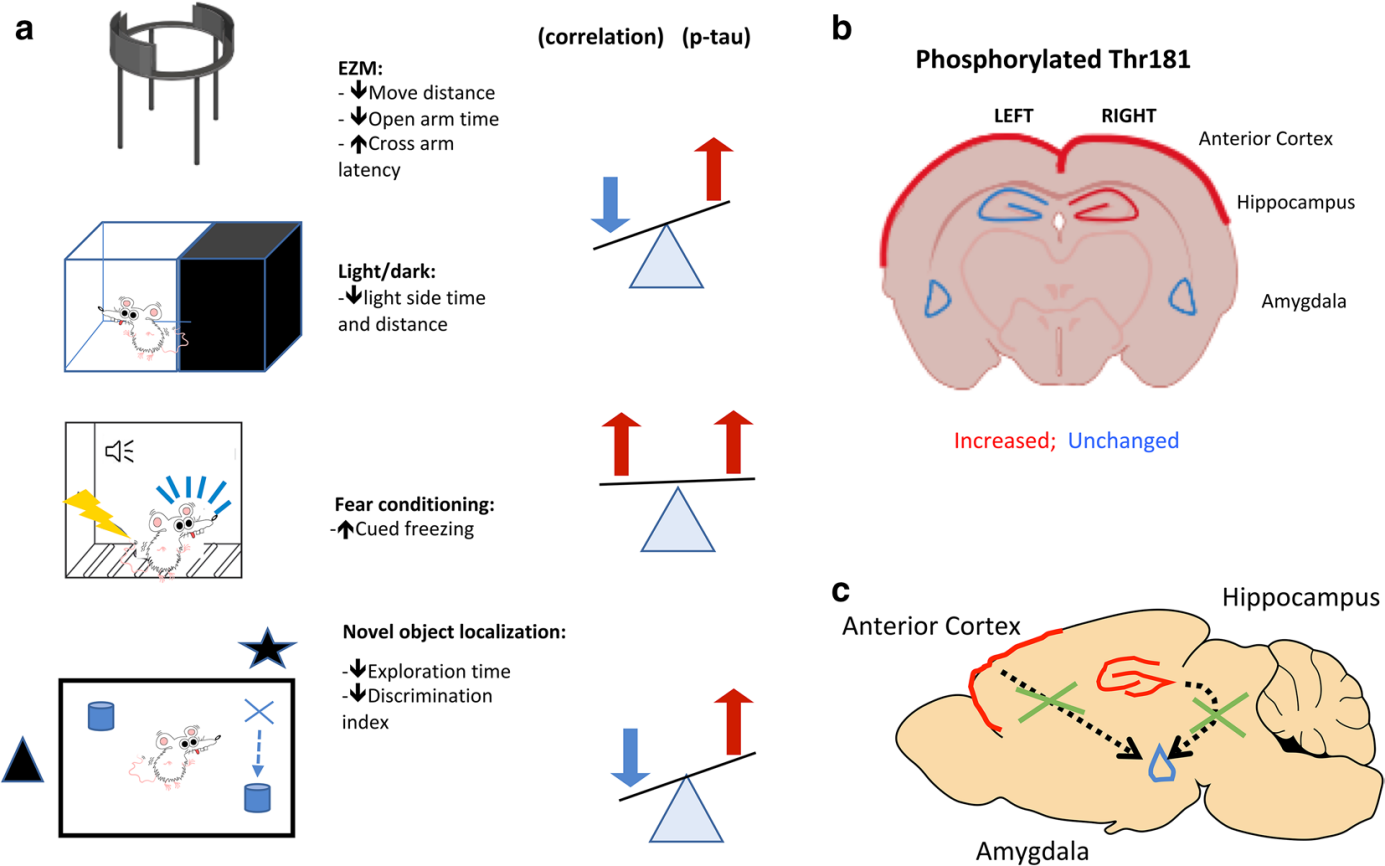
Selected examples illustrating correlations of p-tau Thr181 with behavior. Panel **a** lists behavioral parameters altered in blast-exposed rats at 29 to 35 weeks and correlations with p-tau Thr 181 levels at 40 weeks after blast exposure. Panel **b** shows p-tau Thr181 changes in the indicated brain areas. Panel **c** shows proposed correlation between p-tau accumulation in anterior cortical regions and hippocampus leading to disinhibited amygdala function. Inhibition is indicated by the crosses through the dashed arrows

A number of our observations are consistent with such models and with p-tau playing a role in their molecular basis (Fig. 7). There were strong positive correlations between freezing behavior in the cued phase of a fear conditioning task and p-tau levels in the left and right anterior cortical regions, which in our dissections include the neocortical regions most consistently considered to be part of the rat homolog of the primate prefrontal cortex [54]. The anterior cingulate cortex in our rat model was also a site of abnormal p-tau redistribution into neuronal perikarya [21]. Correlations did not reach statistical significance in the right hippocampus suggesting that anterior cortical function may be a more important determinant of this behavior.

These correlations are consistent with disinhibition models [55, 56, 66] suggesting that p-tau accumulation causing impaired anterior cortical function leads to disinhibited amygdala function without any p-tau elevation or pathology in the amygdala itself (Fig. 7). The fact that in blast-exposed rats, behavioral effects including altered fear learning can be rescued by a drug that stimulates hippocampal neurogenesis further supports the role of hippocampal influences on amygdala function in these animals [79]. It is interesting that one of the few correlations between behavior and total tau was with fear conditioning in the right amygdala where a negative correlation was found between total tau and freezing. The physiological meaning of this and its relationship, if any, to p-tau changes in other regions is unclear. We have observed increases in stathmin 1 expression in the amygdala of blast-exposed animals 9 months after exposure [26]. Stathmin 1 is another microtubule-associated protein that is central in the regulation of fear responses in the amygdala [90]. Further study will be required to determine whether accumulation of phosphorylated tau species in the anterior cortex bears any relationship to stathmin expression changes in the amygdala.

There were no correlations between p-tau levels and responses to acoustic startle or prepulse inhibition. Disordered regulation of these responses are thought to be the basis of the hyperarousal that is a core feature of PTSD [103]. The startle reflex and prepulse inhibition tests depend on the functional integrity of connections within the pons, thalamic intralaminar nuclei, and reticular formation, with forebrain regions likely also playing a role as part of their regulation of complex behavioral states [36, 81, 106]. While we did not measure p-tau levels in these structures, there was no correlation with p-tau levels in the anterior cortical or right hippocampal regions suggesting that tau pathology in these regions is not causing disordered acoustic startle reactions or altered prepulse inhibition.

While not as specific to PTSD as other traits, anxiety is a core-associated feature of PTSD [103]. Tests of anxiety in rodents such as the elevated zero maze or light dark escape task are heavily dependent on anterior cortical and hippocampal regions [81, 93]. Increased p-tau levels in left and right anterior cortical regions as well as the right hippocampus were associated with increased anxiety in multiple measures in these tasks (Table 14).

The laterality of the p-tau accumulation remains puzzling. This effect was most striking in the hippocampus where the right side was always affected but never the left (Table 3). In the anterior cortex, the left side was not affected unless the right was as well. One explanation for this laterality would be if the blast exposure were delivered asymmetrically. Indeed, Turner et al. [101] have described asymmetric p-tau accumulation following both blast and closed head injury models in rats. However, in these studies, the blast exposure and closed head injuries were administered to one side of the head. In our model, the blast exposure is delivered as a straight frontal exposure [1] and there should be no systematic variation in the rat's placement within the blast tube that would cause the right hemisphere to be differentially affected.

We can only speculate that these patterns reflect some laterality of hemispheric function in the rat that causes the right side to respond differently to the initial insult or reflects a feature of how the injury evolves over time. Structural and functional asymmetries in human brain neuroimaging are well known [45] and multiple studies have noted brain asymmetries in blast-exposed veterans in a variety of imaging modalities including diffusion tensor imaging (DTI), functional magnetic resonance imaging (fMRI) and ^18^F-fluorodeoxyglucose (FDG-PET) metabolism [33, 41, 65, 74, 94, 95, 98, 102, 105, 108].

The asymmetrical effects on the right and left hippocampus were particularly striking with all three cohorts showing increased tau phosphorylation specifically in the right hippocampus (Table 4). Differences between the right and left hippocampus in rats are well established [3, 6, 8, 85, 97]. Proteomic profiles differ between the rat left and right hippocampus [85]. Brain-derived neurotrophic factor expression is higher in the rat right hippocampus [3] while during development the expression of the α7 and α4 subunits of nicotinic acetylcholine receptors is higher in the left hippocampus [6]. Insulin-like growth factor-1 receptors show sexually dimorphic patterns of expression [40]. Hippocampal asymmetries also affect serotonergic [8] and angiotensin II [97] modulation of learning and memory in rats. Tau affects stress responses in mice [60] and hemispheric asymmetries have been observed in brain-derived neurotrophic factor and neurotrophic tyrosine kinase receptor type 3 expression in stress-resilient rats [31]. Whether asymmetries in p-tau expression could also be part of a lateralized stress response is unknown.

The results were specific to p-tau Thr181 in that there were no consistent correlations between phosphorylation and behavioral assessments with three other sites (Thr231, Ser396, and Ser404) whose phosphorylation status was not changed following blast [21]. Interestingly, p-tau Thr181 is elevated in plasma-borne exosomes from U.S. military veterans with TBI [49]. Among these TBI veterans, exosomal p-tau Thr181 levels also correlated positively with affective symptoms and PTSD severity [49].

Tau phosphorylation is complex with over 80 potential phosphorylation sites, of which over 40 have been observed experimentally [48, 104]. Thr181 is located in the proline-rich region, which contains multiple Thr-Pro or Ser-Pro motifs, which are targets for proline-directed kinases including glycogen synthase kinase 3β, cyclin-dependent kinase 5 (CDK5), mitogen-activated protein kinase (MAPK) and JUN N-terminal kinase (JNK) [104]. Multiple sites in the proline-rich region are extensively phosphorylated in Alzheimer’s disease [104]. P-Thr181 is deposited in the paired helical filaments found in human NFTs [38] and its level in cerebrospinal fluid is an established biomarker in Alzheimer’s disease as well as a predictor of future cognitive decline [25]. Plasma p-tau Thr181 concentrations are increased in Alzheimer's disease and strongly correlate with amyloid beta-PET-positivity and cortical tau protein deposition as judged by (18)F-flortaucipir PET [99].

## Conclusions

Independent of how blast preferentially modifies phosphorylation at Thr181, this study shows that the laterality and region-specific pattern of its phosphorylation correlate with PTSD-related behavioral traits in rats exposed to repetitive low-level blast. These patterns are consistent with biological models of PTSD suggesting that anterior cortical and hippocampal dysfunction lead to disinhibited amygdala function. These findings provide further evidence for a link between blast injury and tauopathies, which has implications for understanding the relationship of chronic blast-related neurobehavioral syndromes in humans to neurodegenerative diseases.

## Data Availability

The datasets analyzed during the current study are available from the corresponding author on reasonable request.
